# A cross-sectional study examining consideration of self-managed abortion among people seeking facility-based care in the United States

**DOI:** 10.1186/s12978-022-01486-8

**Published:** 2022-08-12

**Authors:** Lauren Ralph, Katherine Ehrenreich, Shelly Kaller, M. Antonia Biggs

**Affiliations:** Advancing New Standards in Reproductive Health (ANSIRH), Bixby Center for Global Reproductive Health, Department of Obstetrics, Gynecology and Reproductive Sciences, University of California, San Francisco, 1330 Broadway Suite 1100, Oakland, CA 94612 USA

**Keywords:** Abortion, Self-managed abortion, Medication abortion, Decision-making, Abortion restrictions

## Abstract

**Introduction:**

With increasing restrictions on abortion across the United States, we sought to understand whether people seeking abortion would consider ending their pregnancy on their own if unable to access a facility-based abortion.

**Methods:**

From January to June 2019, we surveyed patients seeking abortion at 4 facilities in 3 US states. We explored consideration of self-managed abortion (SMA) using responses to the question: “Would you consider ending this pregnancy on your own if you are unable to obtain care at a health care facility?” We used multivariable Poisson regression to assess associations between individual sociodemographic, pregnancy and care-seeking characteristics and prevalence of considering SMA. In bivariate Poisson models, we also explored whether consideration of SMA differed by specific obstacles to abortion care.

**Results:**

One-third (34%) of 741 participants indicated they would definitely or probably consider ending the pregnancy on their own if unable to obtain care at a facility. Consideration of SMA was higher among those who reported no health insurance (adjusted prevalence ratio [aPR] = 1.66; 95% Confidence Interval [CI] 1.12–2.44), described the pregnancy as unintended (aPR = 1.53; 95% CI 1.08–2.16), were seeking abortion due to concerns about their own physical or mental health (aPR = 1.50, 95% CI 1.02, 2.20), or experienced obstacles that delayed their abortion care seeking (aPR = 2.26, 95% CI 1.49, 3.40). Compared to those who would not consider SMA, participants who would consider SMA expressed higher difficulty finding an abortion facility (35 vs. 27%, p = 0.019), figuring out how to get to the clinic (29 vs 21%, p = 0.021) and needing multiple clinic visits (23 vs 17%, p = 0.044).

**Conclusions.:**

One in three people seeking facility-based abortion would consider SMA if unable to obtain abortion care at a facility. As abortion access becomes increasingly restricted in the US, SMA may become more common. Future research should continue to monitor people’s consideration and use of SMA and ensure that they have access to safe and effective methods.

## Introduction

With access to facility-based abortion about to change dramatically in the United States (US), there is growing interest in understanding people’s experiences trying to end their pregnancy on their own outside the formal health care system, often referred to as self-managed abortion (SMA) [1]. Nationally, among those who accessed abortion care at a facility in 2014, 2% reported ever having taken misoprostol or other substances to bring back their period or end a pregnancy [2]. In Texas, a state with many different types of restrictions on abortion, this proportion was higher, at 7% of abortion patients in 2012 and 2014 [3]. Beyond the clinic setting, research indicates that 28% of pregnant people looking for information online about where to access an abortion attempt to self-manage without medical assistance at some point during their care-seeking, and that 7% of US women of reproductive age will attempt to self-manage an abortion outside the formal health care system at some point in their lifetime [4, 5].

Prior research offers insight into some of the factors that contribute to people’s decision to self-manage their abortion. Logistical and practical obstacles, including distance to a clinic, the need for multiple visits, and the cost of abortion and travel, figure prominently [1, 4, 5]. Moreover, interpersonal factors including a desire for privacy; fear of violence, threats, or negative reactions that would affect their wellbeing; and preference for a more natural experience also play a role [6]. Other reasons, including circumstances of the pregnancy and decision-making, are less well studied.

The recent surge in state-level restrictions on abortion which have resulted in clinic closures and increased travel distances to obtain facility-based care have reduced access to abortion in the US [7], and access will be severely restricted as states ban abortion in response to the US Supreme Court’s decision in *Dobbs vs. Jackson Women’s Health*. In this policy context, it is critically important to understand how people might respond if unable to access facility-based abortion care. In this cross-sectional study, we quantify and examine factors associated with whether people seeking abortion would consider self-managing care if unable to access a facility-based abortion.

## Materials and methods

### Data

This analysis uses data from a cross-sectional study designed to develop a new measure of psychosocial burden of obtaining abortion care in the US [8]. From January to June 2019, we recruited participants from 4 abortion facilities located in 3 states (California, Illinois, and New Mexico) with minimal abortion restrictions, but due to their geographic location serve people traveling from more restrictive settings. At each site, clinic staff or a research assistant presented patients in the waiting room with a study flyer and asked if they were interested in participating in a study on “the challenges people face trying to access care to end a pregnancy.” We restricted eligibility to those ages 15 years or older, able to speak and read English or Spanish, seeking an abortion that day, and not pre-medicated with narcotics for a planned procedure.

After being introduced to the study by a research assistant and having patients’ eligibility confirmed, interested participants provided electronic consent, completed a 20-min self-administered, one-time iPad survey in English or Spanish, and received a $30 gift card for compensation. The University of California San Francisco, Institutional Review Board approved this study.

### Measures

We explored consideration of SMA by creating a dichotomous variable to participants’ responses to the question: “Would you consider ending this pregnancy on your own if you are unable to obtain care at a health care facility?” Prior research using this question language demonstrated good comprehension of the phrasing “on your own” and “ending [this] pregnancy” [9]. We considered those who responded, “definitely yes” or “probably yes” to the question as considering SMA and those who responded “probably no”, “definitely no”, or “I don’t know” as not considering SMA.

To identify covariates, we reviewed the literature to identify factors associated with considering self-managed abortion [3–5]. We included questions on sociodemographic characteristics, including age, race/ethnicity, health insurance status, and state of residence, specifically whether the participant had traveled from a different state to access abortion care.

We assessed logistical and practical obstacles to accessing facility-based abortion by asking participants whether any of the following delayed them from obtaining care: finding a place that provided abortions (overall and at their gestation), figuring out how to get to the clinic, finding the money for the cost of care, finding money for the cost of travel, needing multiple visits, parental notification or consent requirements, and travel times. We collapsed responses to contrast those selecting “Yes” vs. “No” or “Don’t know” and then summed the number of delays to create a composite score ranging from 0 (no obstacles) to 7 (all obstacles). To further characterize financial circumstances, we asked participants how difficult it was to “find the money to pay to end the pregnancy.” Those who responded “Very” or “Somewhat” (vs. “Not at all” or “A little bit”) were categorized as having difficulty paying for the abortion. If a participant selected “Very much” (vs. “Somewhat,” “A little bit,” or “Not at all”) to a question that asked how worried they were about other people finding out that they were ending the pregnancy, we considered them very concerned about privacy.

To characterize pregnancy circumstances, we asked participants to select the reasons they were seeking an abortion, which included, but were not limited to, the response options, “I'm concerned about my mental health,” “I'm concerned about my physical health,” and “I'm concerned about the health of the fetus.” We collapsed concerns about maternal mental or physical health, resulting in two binary variables summarizing health concerns for the pregnant person and fetus. Certainty about their pregnancy decision was assessed using a 5-item version of the Decisional Conflict Scale (DCS), which includes statements such as “I feel sure about what to choose,” “I am clear about the benefits and risks of each option,” and “This decision is easy for me to make.” Participants respond to each statement on a Likert Scale ranging from “Strongly agree” to “Strongly disagree.” Consistent with guidance from scale developers [10], DCS scores were summed and then scaled to range from 0 to 100, with lower scores reflecting higher certainty. We assessed pregnancy intention using the question, “Thinking back to just before you got pregnant, how did you feel about becoming pregnant?” Those who “didn’t want to be pregnant then or at any time in the future” were classified as having an unintended pregnancy, while those who wanted to be pregnant “sooner,” “then,” or “later” as intended or mistimed. Finally, we asked participants the date or number of weeks since their last menstrual period started and used this to estimate pregnancy duration in weeks.

### Statistical analysis

We present descriptive characteristics on the study sample, overall and by whether they would consider trying to end the pregnancy on their own if unable to get care at a healthcare facility. We test for differences in the distribution of sociodemographic, pregnancy and care-seeking characteristics by consideration of SMA using Poisson regression models that include a fixed effect for recruitment site. Then, we examine the multivariable association between a reduced set of individual sociodemographic, pregnancy and care-seeking characteristics and likelihood of considering SMA, again using Poisson regression models. We chose Poisson models recognizing that when an outcome is common, prevalence ratios represent a more interpretable and conservative measure of association than odds ratios (ORs) [11–13]. Given missingness on covariates, we re-ran multivariable analyses after multiple imputation of missing values using chained equations. Multivariable results were similar with and without imputation; we present only imputed results. To further characterize the role of logistical obstacles and consideration of SMA, we also describe the proportion that reported each type of obstacle by whether or not they would consider SMA, and assess whether this difference is statistically significant using a Poisson regression model that includes fixed effects for site. All analyses were conducted in Stata 15.0.

## Results

We approached 1092 patients, and 846 (77%) agreed to participate. We excluded 20 due to ineligibility and 2 for iPad malfunctions, leaving 824 eligible people who started the survey. A total of 784 completed at least one-fifth of the survey and represent the multiple imputation sample; 741 reached the primary outcome question on consideration of SMA. The most common reason patients did not complete the survey was due to being called back for their appointment. Participants’ mean age was 27.1 years old (range 15 to 45). Approximately one-quarter of participants were non-Hispanic white (28%), non-Hispanic Black (28%), or Hispanic (24%). The majority (76%) had health insurance, including Medicaid. Nearly one third (32%) of participants lived in another state from the facility where they accessed an abortion. Over one-third (38%) of participants found it very or somewhat difficult to pay for the abortion, and one-third (32%) experienced three or more logistical obstacles to accessing an abortion. Median DCS score was 10 (IQR: 0, 25) and mean score was 14.5 (SD = 15.8); reflecting overall high certainty about the decision. 42% described their pregnancy as unintended, compared to wanted (5%) or mistimed (33%), or not sure (20%) (Table 1).

**Table 1:** Demographic, pregnancy, and care-seeking characteristics of people seeking abortion at study clinics, overall and by whether they would consider self-managing abortion (SMA) if unable to access abortion at a facility

Characteristic	Total sample (N = 741)	Would definitely or probably consider SMA n = 250	Would definitely or probably not consider SMA n = 491	p-value^
Total, n (%)	741 (100)	250 (34)	491 (66)	
	n (column %)	n (row %)	n (row %)	
Age (years)				0.581
15 to 17	33 (4.5)	13 (39.9)	20 (60.6)	
18 to 19	56 (7.6)	20 (35.7)	36 (64.3)	
20 to 24	192 (25.9)	53 (27.6)	139 (72.4)	
25 to 29	217 (29.3)	72 (33.2)	145 (66.8)	
30 to 34	132 (17.8)	51 (38.6)	81 (61.4)	
35 to 45	111 (15.0)	41 (36.9)	70 (63.1)	
Race and ethnicity				0.482
Non-Hispanic White	208 (28.1)	77 (37.0)	131 (63.0)	
Non-Hispanic Black	208 (28.1)	68 (32.7)	140 (67.3)	
Hispanic	179 (24.2)	61 (34.1)	118 (65.9)	
Non-Hispanic Asian or Pacific Islander	46 (6.2)	19 (41.3)	27 (58.7)	
Non-Hispanic multi-racial or other	86 (11.6)	22 (25.6)	64 (74.4)	
Missing	14 (1.9)	3 (21.4)	11 (78.6)	
Health insurance status				0.054
Insured	564 (76.1)	178 (31.6)	386 (68.4)	
No health insurance/Doesn’t know	160 (21.6)	69 (43.1)	91 (56.9)	
Missing	17 (2.2)	3 (17.6)	14 (82.4)	
Difficulty getting money to pay for abortion				0.069
Very	137 (18.6)	55 (40.2)	82 (59.9)	
Somewhat	142 (19.3)	54 (38.0)	88 (62.0)	
A little bit	123 (16.7)	45 (36.6)	78 (63.4)	
Not at all	333 (45.3)	92 (27.6)	241 (72.4)	
Missing	6 (0.8)	4 (66.7)	2 (33.3)	
Lives in a different state from where accessed abortion				0.724
No	507 (68.4)	165 (32.5)	342 (67.5)	
Yes	234 (31.6)	85 (36.3)	149 (63.7)	
Certainty about pregnancy (n = 734)				0.059
Mean score on Decisional Conflict Scale (range: 0 to 100)	14.5	12.7	15.4	
Pregnancy intention				0.020
Wanted	37 (5.0)	10 (27.0)	27 (73.0)	
Mistimed	243 (32.8)	74 (30.5)	169 (69.5)	
Unintended	311 (42.0)	127 (40.8)	184 (59.2)	
Not sure	147 (19.8)	37 (25.2)	110 (74.8)	
Missing	3 (0.4)	2 (66.7)	1 (33.3)	
Seeking abortion due to concerns about health of the fetus				0.667
No	712 (96.1)	239 (33.6)	473 (66.4)	
Yes	29 (3.9)	11 (37.9)	18 (62.1)	
Seeking abortion due to concerns about their physical or mental health				0.127
No	573 (77.3)	182 (31.8)	391 (68.2)	
Yes	168 (22.7)	68 (40.5)	100 (59.5)	
Pregnancy duration				0.967
≤12 weeks	517 (69.8)	174 (33.7)	343 (66.3)	
13 to 19 weeks	107 (14.4)	37 (34.6)	70 (65.4)	
≥20 weeks	107 (14.4)	36 (33.6)	71 (66.4)	
Missing	10 (1.4)	3 (30.0)	7 (70.0)	
Worried about others’ finding out they were ending pregnancy				0.879
Very much	127 (17.1)	46 (36.2)	81 (63.8)	
Somewhat	97 (13.1)	35 (36.1)	62 (63.9)	
A little bit	146 (19.7)	45 (30.8)	101 (69.2)	
Not at all	363 (49.0)	122 (33.6)	241 (66.4)	
Missing	8 (1.1)	2 (0.8)	6 (1.2)	
Number of obstacles that delayed care seeking				0.017
None	305 (41.2)	81 (26.6)	224 (73.4)	
1 or 2	195 (26.3)	81 (41.5)	114 (58.5)	
3 or more	234 (31.6)	87 (37.2)	147 (62.8)	
Missing	7 (< 1)	1 (14.3)	6 (85.7)	

^p-value obtained using post-estimation tests following a Poisson regression model that included a fixed effect for site

When asked if they would do something on their own to try to end the pregnancy if unable to access abortion at a facility, responses included definitely yes (n = 136, 18%), probably yes (n = 114, 15%), probably no (n = 84, 11%), and definitely no (n = 259, 35%), with 20% (n = 148) indicating they did not know [not shown]. Collapsing those who responded definitely or probably yes, one in three (34%) participants indicated they would consider SMA if unable to obtain care at a facility (Table 1).

In bivariate analyses, the proportion who would consider SMA was higher among participants who reported no health insurance compared to those who were insured (43.1% vs. 31.6%, p = 0.054). There were also marginally significant differences in the proportion that would consider SMA by level of difficulty paying for the abortion, ranging from 40.2% among those who said it was very difficult to 27.6% among those who said it was not at all difficult (p = 0.069). Those who indicated that they were seeking abortion due to concerns about their own physical or mental health were more likely to report considering SMA compared to those who did not select this reason (40.5 vs. 31.8%, p = 0.127), and consideration of SMA was higher among those who reported 1 to 2 (41.5%) or 3 or more (37.2%) obstacles that delayed their abortion care seeking compared to those who reported no obstacles (26.6%, p = 0.017). Finally, those who would consider SMA reported lower scores on the DCS, indicating higher certainty about their pregnancy decision than those who did not consider SMA (p = 0.059) (Table 1).

In multivariable analysis, most of these differences remained. Participants who reported no health insurance or didn’t know if they had health insurance were significantly more likely to consider SMA (aPR, 1.66; 95% CI, 1.12–2.44) compared to insured participants. Participants with an unintended pregnancy, compared to a wanted or mistimed pregnancy, were more likely to consider SMA (aPR, 1.53; 95% CI, 1.08–2.16). Compared to those who reported no logistical or practical obstacles that delayed their care, those that reported 1 to 2 obstacles (aPR, 2.26; 95% CI, 1.49–3.40) or 3 or more obstacles (aPR = 1.57, 95% CI: 1.00, 2.47) were more likely to consider SMA. Those who were seeking abortion due to concerns about their own physical or mental health were also more likely to consider SMA (aPR, 1.50; 95% CI, 1.02–2.20) compared to those not seeing abortion for these reasons. While both younger (ages 15 to 19) and older (ages 30 to 34) age groups had elevated likelihood of considering SMA when compared to 20- to 24-year-olds, these differences were not statistically significant (p-value of 0.066 and 0.095, respectively) (Table 2).

**Table 2:** Multivariable association between sociodemographic and pregnancy characteristics and consideration of self-managed abortion

Characteristic	Prevalence ratio	95% Confidence interval	p-value
Age (years)			
15 to 17	2.13	0.95, 4.77	0.066
18 to 19	1.77	0.91, 3.44	0.095
20 to 24 (ref.)			
25 to 29	1.36	0.87, 2.12	0.181
30 to 34	1.64	0.99, 2.70	0.053
35 +	1.43	0.84, 2.45	0.185
Race and ethnicity			
Non-Hispanic White (ref.)			
Non-Hispanic Black	0.94	0.61, 1.45	0.774
Hispanic	0.87	0.55, 1.39	0.572
Non-Hispanic Asian or Pacific Islander	1.41	0.70, 2.87	0.332
Non-Hispanic multiracial or other race/ethnicities	0.54	0.30, 0.98	0.042
Health insurance status			
Has health insurance (ref.)			
No health insurance/Doesn’t know	1.66	1.12, 2.44	0.011
Difficulty getting money to pay for abortion			
Not at all/A little bit (ref.)			
Somewhat/Very	1.30	0.90, 1.89	0.165
Decisional Conflict Scale (DCS) score (continuous, range: 0 to 100)	0.99	0.97, 1.00	0.010
Pregnancy intention			
Wanted, mistimed, or not sure (ref)			
Unintended	1.53	1.08, 2.16	0.016
Lives in a different state from where accessed abortion			
No (ref.)			
Yes	0.74	0.47, 1.17	0.194
Number of logistical delays experienced accessing abortion			
None (ref.)			
1 or 2	2.26	1.49, 3.40	0.000
3 or more	1.57	1.00, 2.47	0.052
Seeking abortion due to concerns about the health of the fetus			
No (ref.)			
Yes	1.75	0.76, 4.03	0.190
Seeking abortion due to concerns about their own physical or mental health			
No (ref.)			
Yes	1.50	1.02, 2.20	0.038

*Ref.* reference; All values obtained from a Poisson regression model that included a fixed effect for study site and imputation of missing values

In descriptive analysis focused on the relationship between consideration of SMA and specific types of obstacles, consideration of SMA was consistently higher among those who reported obstacles related to finding or getting to a clinic. One-third (35%) of those who would consider SMA had difficulty finding a facility that does abortions, compared to one-quarter (27%) of those who would not consider SMA (p = 0.019). Similarly, participants that would consider SMA were more likely to report difficulty figuring out how to get to the clinic (29 vs. 21%, p = 0.021) and needing multiple visits (23 vs. 17%, p = 0.044) when compared to those who would not consider SMA (Table 3).

**Table 3:** Proportion reporting obstacles to abortion care, overall and by whether they would consider self-managed abortion (SMA)

	Full sample	Would definitely or probably consider SMA	Would definitely or probably not consider SMA, or not sure	p-value^^^
Procedure costs (n = 730)	220 (30.1)	81 (33.1)	139 (28.7)	0.221
Finding a place that does abortions (n = 708)	209 (29.6)	84 (35.2)	125 (26.7)	0.019
Travel time to obtain care to end the pregnancy (n = 686)	196 (28.6)	70 (30.2)	126 (27.8)	0.507
Not knowing where to go (n = 730)	201 (27.5)	78 (31.7)	123 (25.4)	0.072
Insurance coverage (n = 730)	193 (26.4)	72 (29.0)	121 (25.1)	0.248
Travel costs (n = 730)	184 (25.2)	68 (27.4)	116 (24.0)	0.316
Figuring out how to get to a clinic (n = 730)	175 (23.9)	72 (29.0)	103 (21.3)	0.021
Finding a place to do the procedure this far along (n = 734)	175 (23.8)	62 (24.9)	113 (23.3)	0.630
Needing multiple visits (n = 730)	141 (19.3)	58 (23.4)	83 (17.2)	0.044
Parental notification or consent requirements (n = 730)	44 (6.0)	14 (5.6)	30 (6.2)	0.741

Non-response is higher to select items because these options were added to the survey several weeks into data collection. All values are row n (%). ^p-value from Poisson regression model that included a fixed effect for study site

## Discussion

In this sample of 741 abortion patients, we find that as many as one in three (34%) indicate that they would consider doing something to end their pregnancy on their own if unable to obtain care at a health care facility. This figure is somewhat higher yet consistent with prior research that finds that 28% of pregnant people searching online for abortion care attempted self-managed abortion during their care seeking process [5]. Thus, our findings reinforce that a hypothetical question asking people what they might do if unable to get care at a health care facility is largely consistent with pregnant people’s actual behavior when they are searching online for a way to access abortion care.

This study was conducted at a time when facility-based abortion, though oftentimes difficult to access [14], was legally protected in the U.S. under the Supreme Court’s 1972 *Roe vs Wade* decision. However, since our study was conducted, the Supreme Court overturned federal protections on abortion and enabled states to enact stricter restrictions or outright bans on abortion. Thus, the hypothetical scenario presented to participants in this study of being unable to access abortion at a facility is now reality for pregnant people living in approximately half of U.S. states where abortion will soon be illegal [15].

In this new policy context, a growing number of people are likely to consider self-managed abortion, as they navigate additional legal risks as well as increased travel distances to the nearest abortion clinic. Research has estimated that with the reversal of federal protections on abortion established in *Roe v. Wade*, 39% of US women of reproductive age will experience increased travel distances to the nearest abortion clinic, with the farthest distance up to 791 miles [16]. Echoing prior research, we find that being uninsured, having difficulty paying for abortion, and facing one or more logistical or practical obstacles that delayed access to abortion care are associated with elevated likelihood of considering SMA [4–6, 17]. Evidence that consideration of SMA is consistently higher for those who report obstacles related to finding or getting to a clinic or paying for care provide indication of what might happen as facility-based care becomes harder to access, and as people are forced to travel further and incur more costs for this care.

New to the literature is our finding that people concerned about their own physical or mental health are more likely to consider SMA. This finding suggests that people with certain pregnancy circumstances may be more strongly motivated to end their pregnancy, regardless of the availability of facility-based care. People with physical and mental health conditions are more likely to experience policy-related barriers accessing abortion [18] and may be particularly vulnerable to the structural barriers imposed on them by restrictive abortion laws, given that these experiences can exacerbate symptoms of stress and anxiety [8, 18–20]. In addition, this study provides some evidence that young people (15–19 years) and people over 30 years are more likely to consider SMA, yet this trend does not reach statistical significance. Further attention to young people is warranted; their frequent exclusion or underrepresentation in abortion-related research precludes in-depth exploration into their experiences and perspectives, and yet they are one of the groups likely to be adversely impacted by restrictions in access.

Our findings have some limitations. First, our main outcome variable asked participants about a hypothetical situation that did not specify specific methods for ending a pregnancy. It is possible that some would consider SMA differently in a future situation than how they indicated in the survey. This may be increasingly true as knowledge and utilization of models of care that mail abortion medications directly to patients increase [21, 22]. Second, while our sample includes people who traveled from restrictive policy settings to access their abortion, study sites were not located in highly restrictive landscapes, where consideration of SMA may be more prevalent among those not able to get to a facility. Furthermore, our sample includes only those who ultimately accessed abortion care at a facility and is missing those who could not overcome obstacles to accessing facility-based care.

Self-managed abortion may become more common as abortion access becomes increasingly restricted. Future research should continue to monitor pregnant people’s consideration and use of SMA, which may give further insight into who is interested in self-sourcing medications or will need assistance traveling to state where abortion remains accessible as clinic-based abortion access is eliminated in regions across the US.

## Data Availability

The dataset generated and analyzed during the current study are not publicly available due to the nature of the IRB approval obtained, but are available in a reduced, de-identified format from the corresponding author on reasonable request.

## References

[1] Moseson H, Herold S, Filippa S, Barr-Walker J, Baum SE, Gerdts C (2020). Self-managed abortion: a systematic scoping review. Best Pract Res Clin Obstet Gynaecol.

[2] Jerman J, Jones RK, Onda T. Characteristics of U.S. abortion patients in 2014 and changes since 2008. 2021. https://www.guttmacher.org/report/characteristics-us-abortion-patients-2014. Accessed 21 Apr 2022.

[3] Fuentes L, Baum SE, Keefe-Oates B, White K, Hopkins K, Potter J, Grossman D (2020). Texas women’s decisions and experiences regarding self-managed abortion. BMC Women’s Health.

[4] Ralph L, Foster DG, Raifman S, Biggs MA, Samari G, Upadhyay UD, Gerdts C, Grossman D (2020). Prevalence of self-managed abortion among women of reproductive age in the United States. JAMA Netw Open.

[5] Upadhyay UD, Cartwright AF, Grossman D (2022). Barriers to abortion care and incidence of attempted self-managed abortion among individuals searching Google for abortion care: a national prospective study. Contraception.

[6] Aiken ARA, Broussard K, Johnson DM, Padron E, Starling JE, Scott JG (2020). Knowledge, interest and motivations surrounding self-managed medication abortion among patients at three Texas clinics. Am J Obstet Gynecol.

[7] New York Times. Supreme Court to Hear Abortion Case Challenging Roe v. Wade. 2021. https://www.nytimes.com/2021/05/17/us/politics/supreme-court-roe-wade.html. Accessed 22 Apr 2022.

[8] Biggs MA, Neilands T, Kaller S, Wingo E, Ralph L (2020). Developing and validating the psychosocial burden among people seeking abortion scale (PB-SAS). PLoS ONE.

[9] Moseson H, Filippa S, Baum SE, Gerdts C, Grossman D (2019). Reducing underreporting of stigmatized pregnancy outcomes: results from a mixed-methods study of self-managed abortion in Texas using the list-experiment method. BMC Womens Health.

[10] The Ottawa Hospital Research Institute. Decisional Conflict Scale. 2020. https://decisionaid.ohri.ca/eval_dcs.html. Accessed 22 Apr 2022.

[11] Zocchetti C, Consonni D, Bertazzi PA (1997). Relationship between prevalence rate ratios and odds ratios in cross-sectional studies. Int J Epidemiol.

[12] Greenland S (1987). Interpretation and choice of effect measures in epidemiologic analyses. Am J Epidemiol.

[13] Barros AJ, Hirakata VN (2003). Alternatives for logistic regression in cross-sectional studies: an empirical comparison of models that directly estimate the prevalence ratio. BMC Med Res Methodol.

[14] Cohen DS, Joffe C (2022). Obstacle course.

[15] Rubin R, Abbasi J, Suran M (2022). How caring for patients could change in a Post-Roe v Wade US. JAMA.

[16] Grossman D, Holt K, Peña M, Lara D, Veatch M, Córdova D, Marji G, Winikoff B, Blanchard K (2010). Self-induction of abortion among women in the United States. Reprod Health Matters.

[17] Myers C, Jones R, Upadhyay UD (2019). Predicted changes in abortion access and incidence in a post-Roe world. Contraception.

[18] Roberts SCM, Berglas NF, Kimport K (2020). Complex situations: economic insecurity, mental health, and substance use among pregnant women who consider—but do not have—abortions. PLoS ONE.

[19] Harris LF, Roberts SCM, Biggs MA, Rocca CH, Foster DG (2014). Perceived stress and emotional social support among women who are denied or receive abortions in the United States: a prospective cohort study. BMC Womens Health.

[20] Biggs MA, Upadhyay UD, McCulloch CE, Foster DG (2017). Women's mental health and well-being 5 years after receiving or being denied an abortion: a prospective, longitudinal cohort study. JAMA Psychiat.

[21] Aiken ARA, Starling JE, Gomperts R, Tec M, Scott JG, Aiken CE (2020). Demand for self-managed online telemedicine abortion in the United States during the coronavirus disease 2019 (COVID-19) pandemic. Obstet Gynecol.

[22] Upadhyay UD, Raymond EG, Koenig LR, Coplon L, Gold M, Kaneshiro B, Boraas CM, Winikoff B (2022). Outcomes and safety of history-based screening for medication abortion: a retrospective multicenter cohort study. JAMA Internal Med.

